# Dissociation and Ionization of Quasi-Periodically Vibrating H_2_^+^ in Intense Few-Cycle Mid-Infrared Laser Fields

**DOI:** 10.1038/srep42086

**Published:** 2017-02-06

**Authors:** Shicheng Jiang, Chao Yu, Guanglu Yuan, Tong Wu, Ruifeng Lu

**Affiliations:** 1 Department of Applied Physics, Nanjing University of Science and Technology, Nanjing 210094, P R China; 2 State Key Laboratory of Molecular Reaction Dynamics, Dalian Institute of Chemical Physics, Chinese Academy of Sciences, Dalian 116023, P R China

## Abstract

Using quantum mechanics calculations, we theoretically study the dissociation and ionization dynamics of the hydrogen-molecule ion in strong laser fields. Having prepared the nuclear wave packet of H_2_^+^ in a specific vibrational state, a pump laser is used to produce a vibrational excitation, leading to quasi-periodical vibration without ionization. Then, a time-delayed few-cycle laser is applied to trigger the dissociation or ionization of H_2_^+^. Both the time delay and the intensity of the probe laser alter the competition between dissociation and ionization. We also explore the dependence of kinetic-energy release spectra of fragments on the time delay, showing that the channels of above-threshold dissociation and below-threshold dissociation are opened and closed periodically. Also, dissociation from different channels is influenced by nuclear motion. The dissociation mechanism has been described in detail using the Floquet picture. This work provides a useful method for steering the electronic and nuclear dynamics of diatomic molecules in intense laser fields.

The irradiation of diatomic molecules by an intense laser pulse can yield very complex dynamics. In past decades, much attention has been given to relevant processes and phenomena in strong-field physics, such as charge-resonance-enhanced ionization (CREI)^1^,^2^, high-order harmonic generation (HHG)^3^,^4^, bond softening (BS)^5^, bond hardening (BH)^6^, above-threshold dissociation (ATD)^7^, and below-threshold dissociation (BTD)^8^. Ionization is a fundamental process in atoms and molecules in intense laser fields, and its probability can be calculated within Ammosov-Delone-Krainov theory based on the strong-field approximation^9^,^10^, within time-dependent density-functional theory^11^, or by solving the time-dependent Schrödinger equation (TDSE)^12^. The ionization dynamics of even the simplest molecule system, H_2_^+^, is more complicated than that of single atoms because of the additional vibrational dimension of the nuclei and the two-center effect. A molecular system undergoes multiple bursts of ionization within half cycles during a laser-field oscillation, in contrast to the widely accepted “tunnel ionization” picture of atoms^13^. Also, double-slit-like interferences have been observed in photo-ionization experiments^14^ on H_2_^+^ and explained theoretically^15^,^16^. In CREI, the ionization rate is maximized for large internuclear distance, exceeding the atom limit by an order of magnitude^1^,^2^,^17^,^18^,^19^,^20^. Litvinyuk and coworkers^21^ claimed to have first observed, experimentally, the elusive double-peak structure in the *R*-dependent ionization rate of H_2_^+^. The rapidity and large spatial distribution of nuclear wavepackets were the main reasons previous experiments failed to observe the double-peak structure. Based on the one-dimensional (1D) Coulomb potential, Qu *et al*. claimed that, theoretically, the ionization and HHG of H_2_^+^ in short intense laser pulses with moving nuclei are substantially different from those in the absence of nuclear correlation^22^. Using the similar 1D model in the non-Born-Oppenheimer (NBO) approach, Nguyen *et al*.^23^ found that the ionization probability initially increases and subsequently decreases as the vibrational level of H_2_^+^ increases.

Another important process in photochemical reactions is molecular dissociation. Extensive studies have been done on H_2_^+^, the simplest diatomic system. If the laser field is intense enough to break the chemical bond, H_2_^+^ displays two fragmentation channels: (i) the ionization channel, i.e., Coulomb explosion (CE),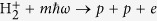, and (ii) the dissociation channel, 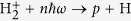. It is well known that BS, BH, ATD, and BTD are important processes in the latter channel. BS describes how the low-lying vibrational wave packets move to the region of large nuclear distances in a strong field, leading to the dissociation of the molecule. On the other hand, BH implies that the high-lying vibrational wave packets are localized in the field-induced bound state^24^. ATD can occur when the nuclear wave packets are excited by absorbing more than one photon. The molecule can also dissociate by absorbing photons with less energy than the dissociation threshold, a process called BTD^25^. The details of these processes have been widely studied in terms of the nuclear kinetic-energy release (KER) spectrum, a generally accepted method^26^,^27^,^28^,^29^,^30^,^31^,^32^,^33^,^34^. The general numerical method for investigating nuclear dynamics involves solving the TDSE within the BO approximation by considering only the lowest or essential electronic states. However, ultrashort and very intense laser pulses make the BO approximation inadequate for treating the interaction between strong fields and molecules, especially H_2_^+^, which contains light nuclei. The dissociation dynamics of H_2_^+^ is demonstrably influenced not only by the ionization of H_2_ but also by the ionization of H_2_^+^ itself when initiated from a neutral target^35^,^36^,^37^.

Since the landmark experiment of Kling *et al*. who observed the electron localization on one of the dissociating nuclei of D_2_^+^ by utilizing a few-cycle phase-stabilized pulse^38^, much attention has been paid to electron localization and how energy is shared between the electron and the protons during nuclear dissociation^39^,^40^,^41^,^42^,^43^,^44^,^45^,^46^. Recently, Wu and his group studied laser-driven electron-nuclear coupling ingeniously in two-dimensional space, achieving one- or two-dimensionally directional dissociation control and electron localization in diatomic hydrogen system^47^,^48^. Most of the directional dissociation controls have been carried out in a 1D space along the direction of laser polarization. In their work, new possibility has been opened to manipulate directional bond breaking of molecules by strong field. Furthermore, they extended experimental measurement to vibrational and orbital resolved electron-nuclear sharing of photon energy for a multielectron CO system even by applying a multicycle laser pulse^49^. These abovementioned studies confirmed the relative influence of electron and nuclear motions. Except for the experiments on electron localization or asymmetric dissociation, long pulses with a full width at half maximum (FWHM) of tens or hundreds of femtoseconds were applied in most studies^50^,^51^,^52^,^53^. As a result, the role of nuclear vibrations has been blurred.

The development of laser technology has allowed the achievement of phase-stabilized few-cycle and mid-infrared laser fields. These advanced technologies have been used to study the dynamics of electrons and nuclei. As early as 1991, the pump-probe technique was used to measure the transient ionization spectra of Na_2_, which probe wave-packet oscillations^54^. The pump-probe scheme has now become a popular method in laser physics. For example, electron localization after the dissociation of a molecule can be controlled by the pump-probe scheme^55^,^56^,^57^,^58^. By tuning the time delay of the pump and probe pulses, and analyzing the ionization^15^,^16^ and KER^28^,^29^,^30^ signals, the vibrational dynamics of small molecules can be determined.

By numerically solving the TDSE in the presence of nuclear-electron correlation, we studied the dissociation and ionization dynamics of quasi-periodically vibrating H_2_^+^ using the pump-probe scheme. The primary aim of this paper is to present an elaborate control of molecular dissociation and ionization, as well as to survey the effect of nuclear motion on ionization and especially on the dissociation of H_2_^+^, using advanced few-cycle and mid-infrared laser technologies.

## Results and Discussion

**Figure 1:**
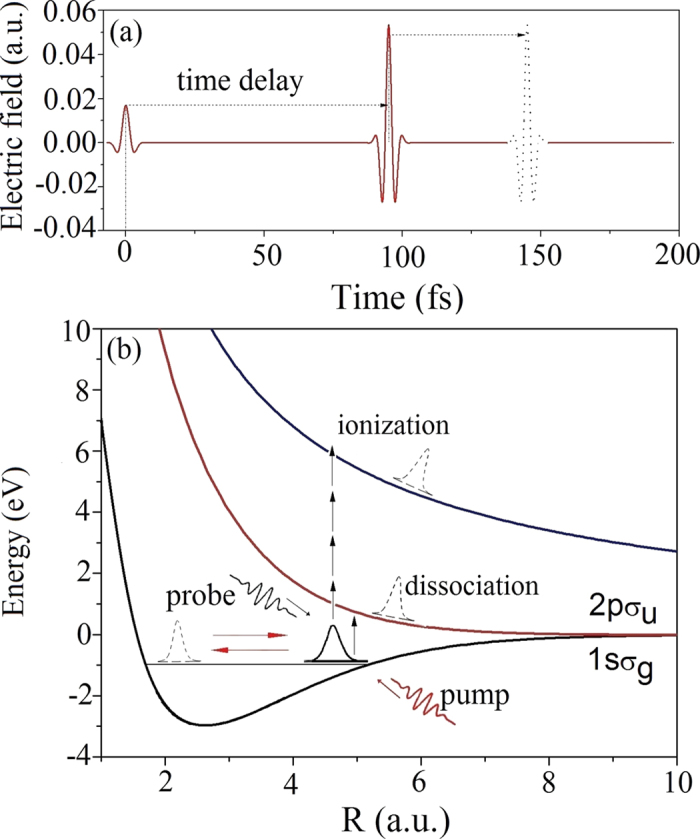
(**a**) Sketch of the pump laser and a time-delayed probe laser. (**b**) Pump-probe scheme with nuclear wave packets in the dissociation and ionization of H_2_^+^.

Figure 1 outlines the laser fields and the pump-probe scheme used in this work. A two-cycle 2400 nm pump pulse with a relatively weak peak intensity of 1 × 10^13^ W/cm^2^ was used to excite vibrations in a nuclear wave packet of H_2_^+^ initially prepared at vibrational level *υ* = 9. Such a weak mid-infrared laser ensures that the molecule do not ionize or dissociate. The chosen initial vibrational state ensured that the internuclear distances *R* within the quasi-periodically vibrating wave packet did not exceed 7 a.u., hence avoiding CREI. After the pump pulse was applied, the whole nuclear wave packet became a superposition of a few vibrational states close to *υ* = 9. This result can be verified using a two-state model that includes only the 1*sσ*_*g*_ and 2*pσ*_*u*_ states. Most of the nuclear wave packet involves the vibrational components *υ* = 8, 9, 10, and 11. Next, an intense time-delayed few-cycle laser pulse triggered the nuclear and electronic dynamics as follows. The wavelength, duration, and peak intensity of the delayed laser were 1600 nm, 13.5 fs, and 1 × 10^14^ W/cm^2^, respectively^59^,^60^. Using this scheme, we could measure the dependences of the dissociation flux, ionization flux, and KER on the time delay, which can be used to probe the dynamics of molecular ionization and dissociation.

**Figure 2:**
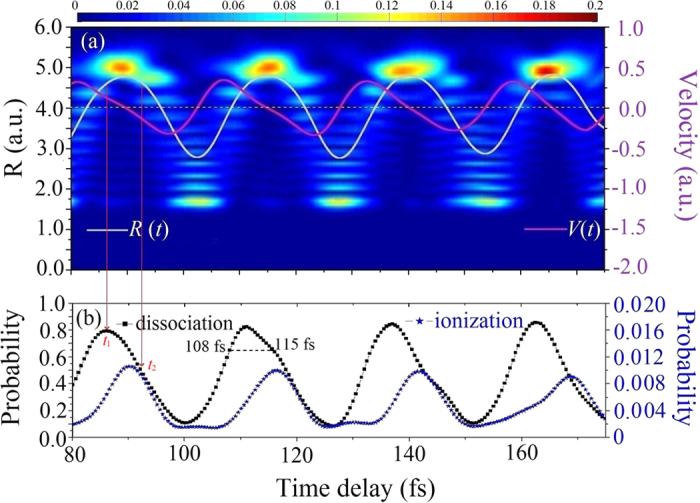
(**a**) Time-dependent density of the nuclear wave packet, displayed as a color map. The white and red lines represent the average internuclear distance and velocity, respectively. (**b**) Dependence of dissociation and ionization probabilities on the time delay. The wavelength, pulse duration, and peak intensity of the delayed probe laser are 1600 nm, 13.5 fs, and 1 × 10^14^ W/cm^2^, respectively.

The time-dependent density of the nuclear wave packet is represented with a color map in Fig. 2a. The time-dependent averages of the internuclear distances and velocities are also shown as white and purple lines, respectively. As shown in Fig. 2b, the dissociation and ionization probabilities modulate with the same period of nuclear vibration. The ionization probability is maximized when internuclear distances reach a maximum value of approximately 5 a.u., where in fact no CREI happens. Interestingly, this trend of ionization probability differs from that reported by Nguyen *et al*.^61^, who found maximum intensity in HHG (proportional to the ionization probability) near the time when the internuclear separation is at the equilibrium value, and the nuclei are moving closer. Because the chosen probe-pulse duration was longer in their calculations (FWHM of approximately 14 fs, i.e., the full duration of approximately 38 fs), the nuclear velocity had a strong influence on HHG. Their results imply that ionization occurs during the falling edge of the pulse, when the internuclear distance exceeds the equilibrium value and the nuclei are moving apart. In the case of the few-cycle probe laser used in the present work, ionization almost occurs around the peak of the probe pulse. Therefore, we conclude that the ionization flux in our scheme is related only to the average internuclear distance when ionization occurs, or rather, to the ionization potential, instead of the nuclear velocity.

**Figure 3:**
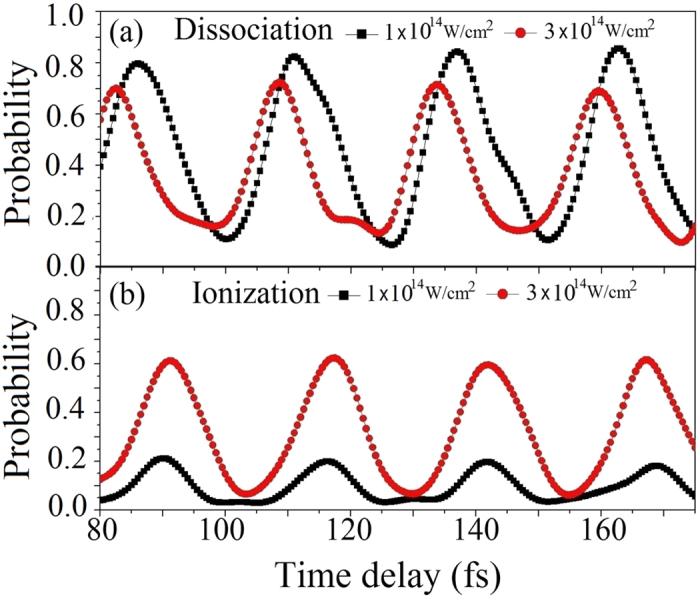
(**a**) Dependence of the dissociation probability on the time delay. (**b**) Dependence of the ionization probability on the time delay. Note that the ionization probability is multiplied by 20 for the 1 × 10^14^ W/cm^2^ peak-intensity data. The other laser parameters are identical to those in Fig. 2.

On the other hand, the maximum of the dissociation probability arrives slightly earlier than that of the ionization probability. Although most of the nuclear wavepackets are distributed around the outer turning point when the average internuclear distance reaches the largest value, the dissociation probability is not the maximum. The maximal dissociation probability arrives when the average internuclear distance *R* is around 4.5 a.u. as marked at time *t*_1_ in Fig. 2. At *R* = 4.5 a.u., the energy difference between the bound and dissociation states is about 2.3 eV which matches the three-photon energy, so the three-photon transition must play an important role and enhance the dissociation^62^. Meanwhile, one should notice that although the average internuclear distances are both around *R* = 4.5 a.u. at *t*_1_ and *t*_2_ in Fig. 2, the dissociation probability at *t*_1_ is much larger than that at *t*_2_. This means that average velocity will also affect the dissociation process significantly. We attribute the much larger dissociation probability at *t*_1_ to the fact that two nuclei are moving away instead of moving close. Thus, we claim for simplicity that the molecules are most likely to dissociate when the two nuclei are moving away at some velocity. The time-delay-dependent dissociation and ionization probabilities with probe intensities of 1 × 10^14^ and 3 × 10^14^ W/cm^2^ are compared in Fig. 3a and b. As the probe-pulse intensity is increased to 3 × 10^14^ W/cm^2^, the peak of the dissociation probability is shifted. We attribute this shift to the strong suppression of the dissociation probability of H_2_^+^ by ionization^50^,^63^. The suppression is much more obvious when the nuclear wavepackets are distributed mostly near the outer turning point. These calculated results demonstrate that the dissociation and ionization dynamics are strongly influenced by the nuclear motion, and therefore the dissociation and ionization of H_2_^+^ can be controlled by applying the pump-probe schemes. For example, when the probe pulse is delayed by 108 fs, the ionization probability is almost zero while the dissociation flux remains large at approximately 0.65. It is therefore worthwhile investigating the KER distribution of dissociative nuclei without significant disturbances being introduced by ionization.

**Figure 4:**
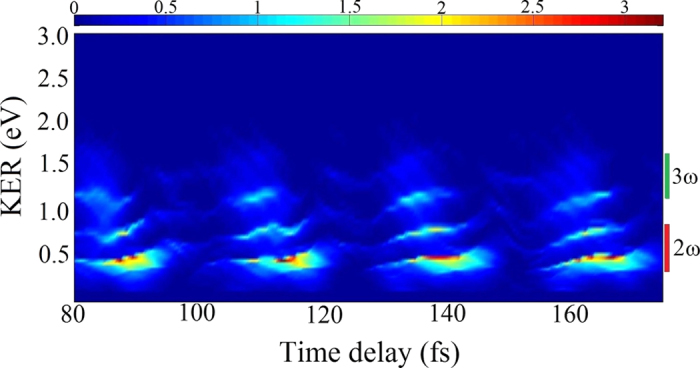
Dependence of the KER spectra on time delay. The red and green bars on the right of the panel indicate the regions of two-net-photon and three-photon dissociation channels, respectively. The laser parameters are the same as in Fig. 2.

**Figure 5:**
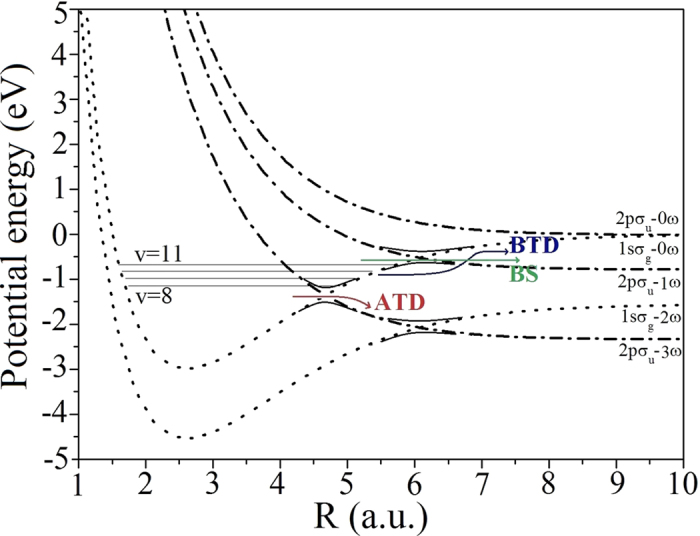
Potential energy curves of H_2_^+^ labeled with the net number of absorbed photons (n*ω*) of wavelength 1600 nm. The solid lines represent the adiabatic anticrossing in Floquet theory, whereas the dotted and dot-dashed lines denote the laser-dressed diabatic potential curves. The horizontal lines represent the vibrational levels, *υ* = 8 ~ 11. The dissociation channels of ATD, BS, and BTD are indicated by the red, green, and blue arrows, respectively.

**Figure 6:**
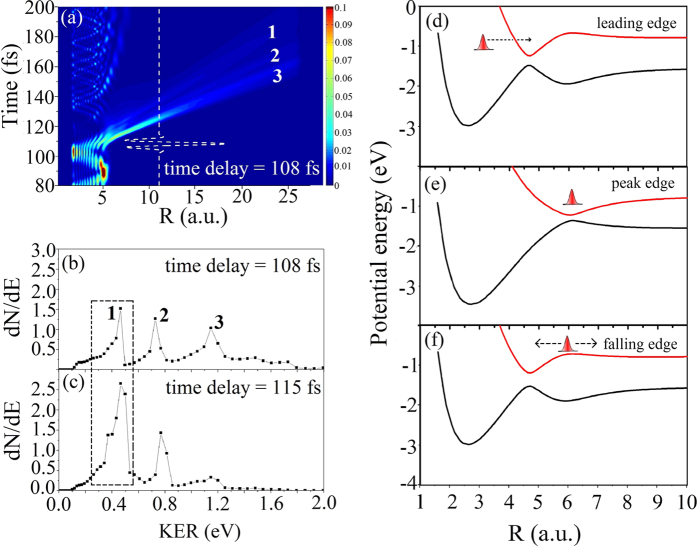
(**a**) Time-dependent density of the nuclear wave packet when a 108 fs delayed probe pulse is applied. The white dotted line represents the electric field of the probe laser. The triple jet-like structure corresponds to the three dominant peaks in (**b**). (**b**,**c**) KER spectra produced when the probe pulses are delayed by 108 fs and 115 fs, respectively. (**d**,**e**) and (**f**) describe BTD dynamics. (**d**) On the leading edge of the probe pulse, a laser-induced adiabatic state is formed and the nuclear wave packet approaches the well. (**e**) As the intensity increases to the main peak, the gap profile changes and the wave packet is trapped in the 3–0 well. (**f**) When the intensity of the probe pulse decreases, the curvature of the laser-induced adiabatic potential near *R* = 6 a.u. changes from upward to downward, so that a part of the trapped wave packet moves to the one-photon dissociation limit. The laser parameters are the same as those of Fig. 2.

The remainder of this work focuses on the KER spectra derived from the dissociation channel for a laser intensity of 1 × 10^14^ W/cm^2^. The dissociative KER spectra, as functions of the time delay, are plotted in Fig. 4. Three dominant peaks appear periodically in these spectra. The large number of dissociation channels makes it difficult to confirm which channel these peaks in the KER spectra belong to. As mentioned above, after the pump pulse, the nuclear wave packet of H_2_^+^ populates the vibrational states *υ* = 8, 9, 10, and 11. The possible photodissociation pathways via laser-dressed Floquet potentials are presented in Fig. 5. In both diabatic (i.e., with crossing) or adiabatic (with avoided crossing) potential curves, these vibrational levels (*υ* = 8 ~ 11) are below the threshold for the one-photon channel. Only the *υ* = 11 state can decay along the one-photon channel by BS. These four states are also above the adiabatic avoided crossing of the three-photon dressed potential. Hence, the nuclear wave packet can dissociate by ATD via (i) the three-photon channel, and (ii) three-photon absorption followed by single-photon release (i.e., a “two-net-photon” channel). The red and green bars on the right of Fig. 4 indicate the regions of the two-net-photon and three-photon dissociation channels, respectively. The time-dependent density of the nuclear wave packets and the KER spectra for the 108 fs delayed probe laser are respectively presented in Fig. 6a and b. The three numbered peaks in Fig. 6b correspond to three jet-like features in Fig. 6a. Peaks **2** and **3** in the KER spectrum arise, respectively, from the two-net-photon and three-photon channels, which have been previously investigated experimentally and theoretically^7^,^64^,^65^.

The mechanism underlying peak **1** is slightly different from that of the other peaks. The jet-like structure labeled **1** in Fig. 6a appears when the probe pulse is almost ended. The BTD process responsible for this effect is illustrated in Fig. 6d–f. The wave packet is firstly trapped in the potential well formed by the three-photon dressed 2*pσ*_*u*_ state and the zero-photon undressed 1*sσ*_*g*_ state (3-0 well). Figure 5 shows the initially populated four vibrational states all being located in this well. At the leading edge of the probe pulse, the nuclear wave packet approaches the laser-induced adiabatic 3-0 well near *R* = 4.5 a.u. The increased intensity modifies the gap shape near the avoided crossing to a larger internuclear distance. The wave packet is thus trapped in the altered 3-0 well near *R* = 6.0 a.u., as shown in Fig. 6e. As the laser intensity falls at the trailing edge of the probe pulse, the laser-induced upper adiabatic potential is no longer a well (upward curvature) around *R* = 6 a.u. but instead curves downward, as shown by the red lines in Fig. 6e and f. A part of the trapped nuclear wave packet then moves to the one-photon dissociation limit. We know from previous studies that peak **3** results from three-photon dissociation, peak **2** from two-net-photon dissociation, and peak **1** from either BTD or two-net-photon dissociation. Furthermore, the dissociation probabilities are almost equal for the cases with delays of 108 or 115 fs (as marked by the horizontal dashed line in Fig. 2b). However, the KER spectra in Fig. 6b and c show significant differences. Because most of the nuclear wave packet is localized at the outer turning point near 4.5 a.u. (Fig. 1a) for the time delay of 115 fs, dissociation from BTD is enhanced; meanwhile, dissociation from ATD via the direct three-photon channel is suppressed, resulting in a higher peak **1** and a lower peak **3**.

## Conclusion

In summary, we explored the dissociation and ionization dynamics of quasi-periodically vibrating H_2_^+^ in an intense few-cycle laser field using quantum-dynamics calculations. Taking advantage of the pump-probe scheme, we obtained the time-delay-dependent dissociation and ionization signals, and also KER spectra. The calculation results demonstrate that the laser-induced dynamics of H_2_^+^ is strongly influenced by the nuclear motion and nuclear-electron correlation. It is certain that the ionization flux is only related to the ionization potential but not to the velocity of the nuclei before the internuclear distance reaches a critical value in CREI. The dissociation of H_2_^+^ is not maximized when the internuclear distance reaches its maximum, which differs from the case of ionization. The hydrogen molecular ion is most likely to dissociate when the two nuclei are moving away at some velocity. As the intensity of probe laser is increased, competition between dissociation and ionization becomes more apparent. By appropriately choosing the delayed time of the probe pulse, we observed three clear peaks in the KER spectra and explained them in terms of both ATD and BTD. These involved processes being opened and closed periodically, depending on the time delay between the pump and probe pulses. As the nuclear wave packet is mostly localized at the outer turning point, the ATD channel is suppressed while the BTD is enhanced. We expect these findings will prove to be useful in studies of ultrafast dynamics in diatomic molecules, exploiting accessible laser technology. They also provide a useful method for steering the electronic and nuclear dynamics of diatoms by means of intense laser fields.

## Methods

All the numerical calculations were carried out using our quantum dynamics program LZH-DICP^66^. Since the used laser is linearly polarized along with the molecular axis, we reduced the quantum dynamics calculations in 1D nuclear coordinate and 1D electronic coordinate (1D + 1D). Atomic units are used throughout unless otherwise stated, therefore, in the dipole approximation the 1 + 1D NBO TDSE for H_2_^+^ can be described as^67^,^68^:


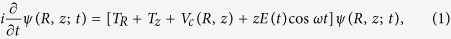


where 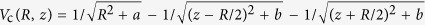 with *a* = 0.0 and *b* = 1.0, 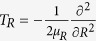, 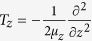, *E*(*t*) is the electric field, *ω* is the laser frequency, *μ*_*R*_* = M*/2 and *μ*_*z*_ = 2 *M*/(2 *M* + 1) are the reduced masses with *M* = 1836.15 a.u., *R* is internuclear distance, and *z* is the electron coordinate with respect to the nuclear center of mass. In the simplified coordinate with a softcore Coulomb potential for H_2_^+^, the computed dissociation energy (*D*_e_) of the ground state and ionization potential (*I*_p_) are different from its real values (*D*_e_ = 3.0 eV from present 1D model vs. *D*_e_ = 2.8 eV from full-dimensional calculations or experiments, and at the equilibrium geometry *I*_p_ = 31.4 eV from present 1D model vs. *I*_p_ = 29.9 eV from full-dimensional calculations or experiments), nevertheless, such differences will not change our main conclusions.

We employ the sine basis functions to define a discrete variable representation for the translational coordinates *R* and *z*. The time-dependent wave function is advanced using the standard second-order split-operator approach with time steps of 0.2 a.u. and 0.05 a.u. for nuclear and electronic movements respectively. To economize on computation time, we take advantage of the disparity in the time scales of the nuclear and electronic motion. Note that the interaction potential includes all the potential energy of the system plus a purely imaginary term to produce an absorbing boundary. In our calculation, *R* is extended from 0.1 a.u. to 30 a.u. with a spatial step of Δ*R* = 0.1 and *z* grid is from −200 a.u. to 200 a.u. with Δ*z* = 0.2.

The “virtual detector” method^66^,^69^ is utilized to detect the dissociation and ionization flux and also to obtain accurate KER spectra. In details, the dissociation flux can be defined as


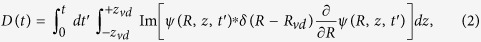


where *R*_*vd*_ and *z*_*vd*_ are the positions of flux analyses for nuclear and electron wave packet. In our calculation, we set *R*_*vd*_ and *z*_*vd*_ to be 20 a.u. and 25 a.u., respectively. Similarly, the ionization flux can be defined as


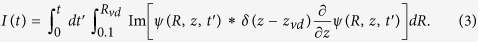


At each time step, the momentum of the dissociation flux passing through *R*_*vd*_ is calculated by


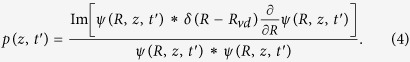


After integration over *z* and through binning of the momentum values for all time, the momentum distribution for dissociation can be accurately determined. Then, the corresponding KER spectra can be obtained. In addition, the time-dependent nuclear probability density in Fig. 6a is obtained by 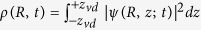. More methodological and computational details are referred to our previous works and the other’s studies on strong fields dynamics and phenomena including KER and HHG^66^,^68^,^70^,^71^.

## Additional Information

**How to cite this article**: Jiang, S. *et al*. Dissociation and Ionization of Quasi-Periodically Vibrating H2^+^ in Intense Few-Cycle Mid-Infrared Laser Fields. *Sci. Rep.* **7**, 42086; doi: 10.1038/srep42086 (2017).

**Publisher's note:** Springer Nature remains neutral with regard to jurisdictional claims in published maps and institutional affiliations.
